# Does a voucher program improve reproductive health service delivery and access in Kenya?

**DOI:** 10.1186/s12913-015-0860-x

**Published:** 2015-05-23

**Authors:** Rebecca Njuki, Timothy Abuya, James Kimani, Lucy Kanya, Allan Korongo, Collins Mukanya, Piet Bracke, Ben Bellows, Charlotte E. Warren

**Affiliations:** Centre for Population Health Research and Management, P.O BOX 1907–00202, Nairobi, Kenya; Population Council, P.O Box 17643–00200, Nairobi, Kenya; Ghent University, B-9000, Ghent, Belgium; Department of Sociology, University of Nairobi, P.O Box 30197–00200, Nairobi, Kenya; Population Council, 4301 Connecticut Ave NW, 20008 Washington, DC USA

## Abstract

**Background:**

Current assessments on Output-Based Aid (OBA) programs have paid limited attention to the experiences and perceptions of the healthcare providers and facility managers. This study examines the knowledge, attitudes, and experiences of healthcare providers and facility managers in the Kenya reproductive health output-based approach voucher program.

**Methods:**

A total of 69 in-depth interviews with healthcare providers and facility managers in 30 voucher accredited facilities were conducted. The study hypothesized that a voucher program would be associated with improvements in reproductive health service provision. Data were transcribed and analyzed by adopting a thematic framework analysis approach. A combination of inductive and deductive analysis was conducted based on previous research and project documents.

**Results:**

Facility managers and providers viewed the RH-OBA program as a feasible system for increasing service utilization and improving quality of care. Perceived benefits of the program included stimulation of competition between facilities and capital investment in most facilities. Awareness of family planning (FP) and gender-based violence (GBV) recovery services voucher, however, remained lower than the maternal health voucher service. Relations between the voucher management agency and accredited facilities as well as existing health systems challenges affect program functions.

**Conclusions:**

Public and private sector healthcare providers and facility managers perceive value in the voucher program as a healthcare financing model. They recognize that it has the potential to significantly increase demand for reproductive health services, improve quality of care and reduce inequities in the use of reproductive health services. To improve program functioning going forward, there is need to ensure the benefit package and criteria for beneficiary identification are well understood and that the public facilities are permitted greater autonomy to utilize revenue generated from the voucher program.

## Background

Results-based financing has emerged as a potentially significant strategy to finance health and other social protection services in the health sector. It is mainly used for expanding the quantity and quality of targeted subsidized health services [1–4]. Results-based financing include initiatives such as output-based aid (OBA), pay for performance, cash on delivery and performance-based financing [5–7]. It includes a range of health initiatives aimed at achieving health targets such as improvement in health outcomes, greater output of specific health services and changes in health related behaviors with increased use of health services by individuals [7–10].

The theoretical context of the OBA or voucher mechanism is found in the basic economics theories of supply and demand with the aim of using market mechanisms to efficiently subsidize health services for individuals who would likely go without the service in the absence of the voucher. The main rationale behind subsiding health care is the inequitable distribution of wealth and health [11]. Voucher programs target disadvantaged populations and contract healthcare providers to see the voucher clients, stimulating both supply and demand for services. The goal of OBA is often to increase access to and use of key services among the disadvantaged (e.g. poor or socially excluded) by offering them a subsidy to help purchase health services, and preferably by being able to choose a provider from among a number of alternatives [9, 11, 12]. Voucher programs have the potential to reduce gaps in equitable healthcare utilization by subsidizing low-income individuals’ purchasing power [11]. Voucher programs provide an economic incentive to accredited facilities by reimbursing them for services offered [13]. By doing so, the program can stimulate a market for the services and may generate greater competition between providers and indirectly motivate improvements in quality of services.

OBA programs provide incentives to clients and healthcare workers and subsidize specific health care services based on pre-determined prices [7, 11, 14–16]. When a client needs the services, s/he redeems the voucher for the specified service at one of the accredited facilities. The provider is then reimbursed service cost or paid an incentive upon submission of a claim and supporting evidence to the voucher management agency (VMA).

In Kenya since 2006, the OBA program funded by the German Development Bank (KfW) and the Government of Kenya delivers targeted subsidies for safe motherhood (SM), family planning (FP) and gender-based violence recovery services (GBVRS) to promote quality care and maximize healthcare utilization by people living in poverty. The SM and FP vouchers are sold through distributors to poor women in rural districts and low-income areas of Nairobi for a highly subsidized price, while the gender-based violence recovery services (GBVRS) vouchers are provided for free in GBVRS accredited facilities regardless of socio-economic status. The program was launched to generate health sector experience in targeting, accreditation, claims, reimbursement and quality for the proposed National Social Health Insurance Fund. The program has operated in Kisumu, Kitui, Kiambu and Kilifi counties, and Korogocho and Viwandani informal settlements in Nairobi. These sites are seen as representative of different regions of Kenya with poor populations found in both urban and rural settings.

Globally, voucher programs have been associated with increased skilled birth attendance, uptake of long-acting family planning methods, reduction in out-of-pocket expenditure, improved quality of care and improved access to care [17–21], but little is known about the facilities’ response to OBA programming. Drawing from interviews with healthcare providers and facility managers, this study assesses how well they understand the OBA voucher program, their attitudes toward the program, and their observations on the program’s benefits and challenges.

## Methods

The paper draws from qualitative data collected in 2010 as part of a larger quasi-experimental design aimed at evaluating the impact of the Kenyan OBA voucher scheme on increasing access to, and quality of, selected reproductive health (RH) services [22]. These data were derived from in-depth interviews (IDIs) with facility managers and healthcare providers from program sites in Kitui, Kiambu, Nairobi, and Kisumu. The IDIs took place alongside quantitative health facility assessments and population surveys aimed to measure the impact of the voucher program on access to health facilities and quality of services. Details of the larger evaluation and population surveys are discussed elsewhere [22].

The in-depth interviews aimed at gaining a deeper understanding of the perceptions and priorities of the health care providers regarding the voucher program. The interview guide was amended appropriately after pre- testing the tools. The in-depth interview guide focused specifically on: (i) technical knowledge of services offered using the voucher (ii) attitudes towards the voucher program iii) benefits and (iv) challenges of the voucher program, processes and the approach (v) and perceptions on program implementation. A total of 69 interviews with facility managers (*n* = 30) and healthcare providers (*n* = 39) working at 30 OBA accredited public, private, and faith-based facilities were conducted. These facilities were randomly sampled from the 54 voucher accredited facilities selected to participate in the first phase of the Kenya OBA program. We planned to interview the facility manager and healthcare providers working in the reproductive health units in the study facilities. However, in some facilities the facility managers were not present on the days of the interviews, therefore interviews were only conducted with the healthcare providers directly involved with reproductive health or maternal and child health services. Overall the study interviewed 46 females and 23 males from facilities ranging from dispensaries to hospitals.

Table 1 presents the distribution and composition of healthcare providers and managers.

**Table 1:** Distribution and composition of service providers and managers

Respondent	Health sector	Dispensary level	Health center level	Hospital level	Total respondents
		Level of facility			
Service provider	Private	1	4	1	6
	Public	4	9	14	27
	Mission	-	2	8	10
Facility health Manager	Private		2	3	5
	Public	3	4	9	16
	Mission	-	2	3	5

The IDIs were tape recorded and transcribed into a Microsoft Word file. The transcribed texts were then transferred to NVIVO 10 qualitative data analysis software. Qualitative data analysis was done by two researchers to ensure reliability in the coding and results. The study hypothesized that a voucher program would improve reproductive health service delivery and access to target population. A combination of inductive and deductive analysis was conducted based on previous research and project documents.

Following coding, a full list of themes was available for categorization within a hierarchical framework of main and sub-themes. The thematic framework was then systematically applied to all of the interview transcripts. We looked for patterns and associations of the themes and compared and contrasted within and between the providers and facility managers in different regions, sectors and levels.

Ethical approval for the evaluation was granted by Population Council’s Institutional Review Board (IRB) No. 470 and Kenya Medical Research Institute (KEMRI) SCC 174. Informed consent was obtained prior to all interviews that were conducted in settings that ensured privacy and confidentiality. Participants were informed that they could withdraw from the research at any time. Data collectors were trained on ethical conduct.

## Results

### Knowledge of vouchers benefit package and technical competence for providing the services

Our findings demonstrate different levels of awareness among healthcare providers and managers in relation to the vouchers benefit package across the facilities. Overall, facility managers had better awareness of the FP and SM services package compared to the healthcare providers. However, facility managers from the dispensary level demonstrated poor knowledge of postnatal care (PNC) services provided through the vouchers. Healthcare providers working in the dispensaries also demonstrated low levels of knowledge of PNC as well as long acting and reversible contraception (LARC) and permanent methods (PM) (LARC-PM) as a benefit package of the voucher. One healthcare provider explains as below

*“They should train on the OBA program procedures. The first thing; the kind of services that they would want us to give, what the card covers for so that we don’t keep on messing then what level of service does OBA cover; where can it cover, where would it people involved. I wish they could also come in and sponsor some trainees to help in improving such as the gender sexual violence”. (IDI, Healthcare provider)*

There were also differences in the knowledge of services covered under the voucher program. Health healthcare providers from the private and public sector demonstrated better knowledge of the SM and FP voucher package compared to the mission-based facilities. There was poor knowledge of GBVRS voucher benefit package across all facilities, levels and sectors. The FP voucher was not well understood by some facility managers and providers, with some stating that it covered both short and (LARC-PM) methods while majority did not know what services were covered by the GBVRS voucher.

There is a felt need by providers and managers for training in GBVRS, (LARC-PM) FP methods and reproductive health cancer screening. The following extracts demonstrate the need for more clinical training;

*“We have not yet been trained into handling those sorts of things [gender based violence] so we refer it to xxxx hospital”. (IDI, healthcare provider)*

*“The training should provide updates on the training of implants and the IUCD and all these OBA …sterilization, voluntary surgical contraception”. (IDI, Healthcare provider)*

*“We need training on GBV which they promised to come and hold training with us. We still have no clear understanding of it. We have patients who come since there are cases but when we refer, we break confidentiality since we don’t have knowledge and guidelines on how to handle them [GBV cases]”. (IDI, Healthcare provider)*

*“They need to include other things – for example, train on counseling on how to handle rape care, which has been challenging. Also, train on the P3 forms [post-rape forms], which are not well known….also train on long-term family planning methods and more nurses should be trained on emergency contraception because they are few who know”. (IDI, healthcare provider)*

In particular, providers working in outpatient health centers, dispensaries and faith-based facilities outside of Nairobi were unaware of the benefits of the GBVRS voucher or how one obtains the voucher if needed. The GBVRS voucher was restricted by program design to Nairobi and only a subset of facilities were contracted to provide the service.

Our findings demonstrate that the most felt benefits of the voucher program by facility managers and healthcare providers were that (i) the voucher program was a profitable business model that promotes competition and general improvement of quality of care (the processes, structures and outcomes); and (ii) the vouchers program plays a positive role in reducing inequity and improving access to maternal, newborn and other reproductive health services.

#### Voucher program as a profitable business model

Across the different study sites, facility managers reported a primary benefit of the program to be the reliable source of revenue it provided. Managers at private and mission facilities reported the source of income as the biggest benefit from the program. Although facility managers in the public sector reported the income from the program as a benefit, majority of the health facility managers expressed disappointment with being unable to use the funds to improve their services due to restrictive guidelines on use of the finances from the MOH. A facility manager had this to say;

“*The advantage to the facility is, one; they are getting another source of funds which is coming to boost the facility’s upkeep and standards. Once that money is projected to the maternity, we are able to acquire things, which we would otherwise wait for the government to deliver and it might take too long. By it being a source of funds to the facility it’s assisting the facilities.” (IDI, Public Facility manager)*

*“It’s a challenge because if there is that competition and we are not offering that standard given by other facilities, so there is competition for clients so we need to improve our facility even more.” (IDI, Public Healthcare provider)*

Majority of health providers and managers working in private facilities felt that the extra revenue ensured availability of supplies, drugs, equipment, improving client comfort through the provision of meals, accommodation, sanitation and ensuring cleanliness and proper hygiene. In the private sector, managers reported that the investments were aimed at attracting more clients and so they concentrated more on expanding the facilities by building more wards and employing more staff. In faith based facilities, majority of the facility managers reported that they focused more on improving the clients’ comfort by increasing number of beds, improving meals, and provision of warm water. Although most health facility managers from public facilities reported inability to utilize reimbursed funds, a few public facilities were able to utilize reimbursed funds. These were mainly higher level facilities (district and county level) which were directly managed on the day to day basis by the county/district health management teams. These facilities utilized the funds on renovating existing structures and purchasing supplies such as curtains, patients’ personal effects including sanitary pads, purchase of medical equipment, supplies and drugs and improving community health education programs. The funds had also been used to improve on the job training for staff especially on reproductive health and medical complications and to conduct community-based health education at churches, chiefs’ barazas (meetings), and community groups and through the media. The quotes below demonstrate how the income earned was utilized;

“*As for xxxxx, we’ve been able to paint the maternity wing, to provide hot showers, offering bath towels, basins and slippers, soap. We are also able to prepare food for our clients and to purchase a room heater for the delivery. We have also purchased a resuscitator for the babies. Staff motivation; we are able to provide lunch, training which we are able to sponsor.” (IDI, Private Healthcare provider)*

*“I think quality has improved in many ways because the revenue from OBA caters for general services that we give such as making sure that the right infrastructure is available, that the right services are offered in terms of quality. The revenue from the OBA has been directed towards the welfare of the clients, the welfare of the facility, the infrastructure within labor ward and even the welfare of staff. For example staffs take tea and better feeding for maternity mothers. Initially we didn’t offer tea continuously for mothers but at least now there’s tea available in labor ward and we are also preparing porridge, which is always available for the mothers.” (IDI, Public Facility manager)*

Majority of health facility managers in the private sector compared to the public sector reported using the revenue to improve customer service, communication with clients, and staff friendliness to ensure client satisfaction as demonstrated by the excerpts below;

*“Recently there is improvement in client-staff relationship. The staffs have tried their best to improve themselves so that they may attract many clients, the more you are good to them the more you attract them and they will bring in the voucher and you will be able even to help your hospital expand. The voucher has now helped the providers to realize need of the clients so that they don’t have to quarrel the clients, be friendly, they have to encourage them to come to the facility.” (IDI Private Facility manager)*

*“If you just go to the wards you will see the improvements in the facility, for example the curtains of the delivery room and ante-natal room are matching with the bed covers, something you would not expect from a government facility. The privacy has improved a lot because now you will find those beds that used to be exposed they are now partitioned with curtains to improve the privacy of the mother, whereby they are even able to stay together with the husband. Before the program there was lack of privacy” (IDI, Public Health Manager)*

“*I have seen facilities that just used to look like ordinary GOK (Government of Kenya) facilities but with the OBA you go there you will not believe it, you will just think you are in a private facility. And when you find the services that are offered to the mother they quality of the care. For example you find the midwife who has conducted the delivery is motivated with 500sh which goes a long way in encouraging the midwife to continue welcoming those who come for ANC promoting their facility even taking them to the labour to see and all that.” (IDI, District Public Health Nurse*)

A majority of health workers at the private facilities reported that reimbursed funds were used for staff motivation in way of compensation for overtime and improvement of working conditions. For example, food and beverages were offered to healthcare staff during working hours.

*“The quality is nice because they use the reimbursed funds to improve the quality of care, for example you find the midwife who has conducted the delivery is motivated with 500ksh given to her by the facility which goes a long way in encouraging the midwife to continue to welcome those who come for antenatal clinics thus promoting their facility even taking them to the labor to see and all that.” (IDI, District Public Health Nurse)*

*“With the facilities offering the OBA program…, rarely do I get frustrated when I conduct a delivery and then I’m threatened by the boss that “you have conducted that delivery, who will pay for it?” Nowadays we don’t have that; we know the voucher will cater for it. But there before we used to get that threat.” (IDI, Healthcare provider)*

#### Reducing inequity and improving access to maternal and newborn services

Providers from private facilities reported increased number of client’s, especially poor women who would otherwise not afford to seek their services. Overall, majority of health providers and managers felt that the voucher program had increased access to and use of reproductive health services, especially skilled birth delivery, among the poor. Most providers cited improved of children as an unintended contribution of the program due to reduced health financing burden.

*“The majority of the people in the community are poor, and if they are able to access these vouchers, it is helping them because they could not have had access to a health facility like here because of money issues, but now they are able to get services because of these vouchers.” (IDI, healthcare provider)*

*“Because we are able now to capture all mothers especially the poor mothers who never come to facilities. If a mother has come for ANC we give them the health talk, we encourage them, and there are those mothers who get precipitate labor maybe at night they deliver at home, not intentionally. So they are able to come the following day for their check up and the checkup of the baby and also family planning.” (IDI Facility manager)*

*“We are seeing the family planning uptake is also high because once you follow the client well in the antenatal period they come even post-delivery. They are able to follow up their clinics [appointments] well.” (IDI, Healthcare provider)*

*“I think with the coming of the OBA, the number of clients who are seeking especially deliveries in hospitals has increased and there is an increase in the reimbursement, whatever they pay. We are also able to improve our services, like we are able to buy new bed sheets, new beds; new delivery beds and we are able to make the wards a bit more comfortable. We have put tiles in the wards and put curtains so that they feel comfortable.” (IDI, Healthcare provider)*

*“Some babies who used to miss the first vaccinations which are very vital and now they normally get them. Like now the babies who are being born are protected from the word go. Before the Voucher program you could get a baby at six weeks without a BCG, but currently you can’t get unless somebody is very ignorant. Mortality rate in the community has gone down if I may say so and diseases which normally get children who are not protected; like now mostly our children are protected from TB.” (IDI, Healthcare provider*

### Facility managers and providers’ perceived challenges with the voucher program

Facility managers and providers identified the key challenges they experienced with the voucher program to be mainly: (i) the working relations of the health facilities and the VMA including some aspects of the program functioning designs (ii) effect of vouchers on the workload (iii) existing challenges with the public health systems

#### Complex reimbursement and claims process

Although most managers interviewed reported ease in preparing the required reports by the voucher management agent (VMA), those in the rural areas reported dissatisfaction with the fact that they had to deliver the reports monthly to the VMA field managers who were based in towns. Health workers in the rural areas described their facilities as being understaffed, therefore, sending one of the staff to deliver the report created a problem of further less providers in the facility. They also felt that lack of accessible roads created problems in delivering the reports. Some providers cited lack of vehicles or transport. The majority of health facility managers observed that delay in reimbursement was a great challenge. Such managers felt that electronic reporting should be adopted by the VMA, and that overall communication and provision of feedback to the facilities needed improvement by the VMA. In addition, use of standard operating procedures on claims and reimbursement could improve communications between providers and VMA. Further such rural facilities’ managers felt that the VMA failed to provide prompt feedback for claims against services offered and sometimes there was no feedback on claims that were declined. Such providers and facility managers felt the nature of the relationship with the VMA was poor. However, health facility managers and providers in most urban areas felt that the relationship and support provided by the VMA was adequate and most managers were satisfied with the reimbursement process. The excerpt below demonstrates the support that health workers expressed.

“*We need to have some of the computer machines that will facilitate us prepare and send the required reports”. (IDI, Healthcare provider)*

“W*e can benefit more from the training together with my fellow colleague so provide training for those who were not trained. I think just about the procedure that should be followed so that at the end of the day we can be reimbursed without any question”. (IDI, Healthcare provider).*

#### Effect of vouchers on the workload

Facility managers and providers in public and private facilities reported different effects of the voucher program on workload (in terms of increasing patient flow). Majority of facility managers and providers in public facilities reported that the voucher increased their workload, in light of the fact that the government had not deployed new staff to cater for increased utilization. Most providers in public facilities reported that with increased workload they felt overwhelmed. However for public facilities that lacked structures that facilitated continuous improvement of quality of services offered, the client load reduced due to increased competition from accredited facilities.

*“Competition from the private [sector] is affecting other facilities for example because we have the GOK (Government of Kenya) facilities that are not accredited so [at] those ones the deliveries come down. We find ourselves explaining to the provincial managers who expects us to do better than that but of course we tell them that when the OBA came in the mother will always choose the best place to deliver in. And most of the time it happens to be the private facilities”. (IDI, Facility manager)*

“*Like now you see I’m alone. Today I had to conduct a delivery and I have a queue here. The delivery I’m conducting is for a voucher user and the antenatal mothers who are seated here are also voucher users. So I either had to delay the delivery, which cannot be delayed but the mothers seated here had to delay. So lack of staff is affecting us”. (IDI, Healthcare provider)*

#### Existing challenges with the public health systems

Most managers from dispensaries and health centers in the public facilities had the view that that the referral mechanism needed to be improved. They describe the situation as difficult due to the lack of ambulances or taxis, especially in the remote areas and when available they took time to provide services or the ambulances had mechanical problems.

*“I think most of the challenges are in referral, we may call the ambulance and they say they are sending the ambulance, and you know from xxxx to here is xxx Kilometers. The roads are terribly bad, there before when the roads were better they could take 45 min… [if there is] fetal distress or this mother is bleeding or there is a cord prolapse, you know that one now, it worsens the whole thing”. (IDI, Health Facility Manager,)*

In public facilities some providers and managers reported that at times they were unable to offer services due to lack of drugs, supplies and equipment and the problem of understaffing. Some managers in the public health facilities observed that voucher clients complained of lack of privacy and sharing of beds.

“*There is overcrowding especially if the space is not big and if it is not possible to expand. Because like now you find clients are sleeping two because they have only one postnatal room. So when they deliver and they are many together with the others we don’t have separate beds to keep them so you make them just share. So she may feel she has not been given the best because she has a voucher”. (IDI, Healthcare provider,)*

Some public facilities managers felt that they were not able to improve many aspects of the services offered because of the inability to utilize reimbursed funds.

“*Initially we didn’t have a problem but from March this year there was this circular from the Ministry of Health that we should have one joint account so we are not able to access the money direct. So we are taking too long to get the money so we are not able to cater for our clients the way we are supposed to because of these government bureaucracies and yet there is a lot of competition from other facilities”. (IDI, Facility Head Nurse)*

## Discussion

This paper explored the views of facility managers and providers on the Kenya voucher or OBA program. The study findings identify factors affecting program function as well as gauged supply side perceptions of the voucher program effects on improving access and utilization of reproductive health services. Our study demonstrates a wide variation in providers’ knowledge and awareness of the SM, FP and GBVR voucher services across the different health sectors and facility levels. The GBVR, FP and the postnatal care (PNC) component of the SM voucher are least known by health providers from dispensaries and health centers. In addition the FP is least known by health providers working in faith-based facilities. However, the SM vouchers were most well-known across all sectors and health care levels while the GBVR voucher was the least known. The low levels of awareness of FP vouchers in faith-based facilities could be attributed to the fact that most of these facilities do not support FP initiatives. This variation in knowledge appears to have affected the functioning of the OBA program design at the lower level facilities. For example, PNC services were sometimes not offered as part of the SM vouchers as providers did not know that PNC is covered by the voucher. In some facilities short term FP methods were provided under the voucher scheme although the program does not cover such services and such claims were further not reimbursed. The poor awareness of GBVRS voucher was accompanied by reported poor technical knowledge on the provision of GBVRS by majority of providers and managers interviewed. The variation in the knowledge and awareness of the benefit package of the different reproductive health vouchers could be attributed to limited training on specific RH issues as well as staff transfers and movements in and out of voucher accredited facilities. This poor level of awareness of the vouchers benefit package could also be attributed to: the lack of training updates on the voucher design and functioning; and existing gaps in program implementation with respect to marketing the voucher to health providers in accredited facilities. This points out to the need to improve information sharing between the VMA and accredited facilities on the key program designs. The knowledge gaps in relation to FP methods covered within the voucher program among mission facilities and lower level facilities could also be explained by the fact that, long-term FP methods were not offered at catholic (mission) and out-patient facilities; and majority of lower level facilities offer out-patient services only. At the dispensaries and health centers which mainly provide outpatient facilities, there was limited staff who would, for example, insert an IUD or implant. Still, the Catholic-based facilities could not provide the FP services due to their religious beliefs and values. These variation on knowledge and use of the SM, FP and GBV vouchers has also been demonstrated among targeted clients in the same program setting with a similar trend of better knowledge and use of SM and least knowledge and use of GBVR vouchers [23]. This variation of knowledge and use of the vouchers in both health workers and among the targeted clients could be indicative of the need to improve both facility and community sensitization of the voucher program and the benefit packages.

Our findings demonstrate poor skills, competence and knowledge to offer (LARC-PM) FP and GBVRS among providers especially those from health centers and dispensaries. This has implications on the quality of services offered. This calls for an urgent need to build providers capacity in all OBA accredited facilities to provide basic GBVRS and long acting and reversible contraception (LARC) and permanent methods (PM) (LARC-PM) Methods. There is a need to avail the recently updated national guidelines on management of sexual violence in Kenya and the national FP guidelines to all healthcare providers. Several other capacity building opportunities exist such as medical schools, nursing programs, clinical officers and laboratory technicians reviewing their curriculums to endow health workers with the relevant knowledge and continuous on job refresher training.

Although the OBA program was implemented to run within the existing health system, the public facilities performance was affected by this assumption since the inherent health system challenges in Kenya include lack of in-service training to update provider’s knowledge and skills. Overall, the low technical knowledge in key services provided within the voucher program highlights gaps in quality assurance that need to be strengthened. The effect of the voucher on the client load has implication on the implementation of the program. The private sector is able to adapt well by increasing the staffing levels and improving structures to accommodate the increased client load. However the increased workload in the public sector as a result of increased demand clearly indicates significant pressure on the current health system structure in government facilities. Care should be taken to ensure such facilities do not compromise the quality of services offered such as the rights and dignity of the clients in an attempt to meet the increased demand.

Majority of the facilities reported that they were affected by the delay of the VMA to reimburse the claims on time. Since the process and the benefit packages were not well understood, facilities submitted claims that were not reimbursable. Secondly, the VMA sometimes failed to provide feedback on reasons claims were not payable or for possible reason for delay in reimbursement further generating dissatisfaction with the claims and reimbursement process. It was evident that OBA training were non-continuous and paired with regular provider transfers and this impacted negatively specifically on the claims and reimbursement system. This resulted to poor working relationship of the VMA and some accredited facilities.

An important aspect in project implementation is communication and feedback between the various actors. Communication within the program between the providers and VMA appears to have been inadequate especially in rural facilities. Providers felt that automated systems and quicker reporting and reimbursement systems are key to better functioning of the program. If the OBA program is to function more efficiently there is need to explore the opportunity of decentralizing the claims process so as to offer more prompt services and feedback. There is also need to automate the claims and reimbursement system to reduce time and other expenses such as transport involved in claiming for the services provided.

The OBA program was affected by the existing health system challenges within the public sector including lack of drugs, understaffing, lack of equipment and supplies, poor referral systems, poor quality of care such as bed-sharing and poor hygiene. There is dire need to strengthen the public sector health system, and there are opportunities with the voucher program to utilize reimbursed funds to improve the facility especially the public sector. However, care should be taken to ensure facilities are not over dependent on the voucher reimbursed funds for expansion and management. Over dependence of the facilities on reimbursement from voucher programs poses a concern regarding the sustainability of the facilities in the absence of the voucher program. There is also an opportunity for the OBA program to strengthen supply chain management of supplies and commodities within accredited facilities to reduce stock outs. However, to improve such systems in the public sector, there is a need to advocate for change in policy environment such as allowing the public sector to utilize the reimbursed funds for expansion and improving the services provided. Further the OBA program is facing challenges in the rural areas associated with poor road network, limited transportation affecting access and the referral system. Although improving road infrastructure is beyond the scope of the program, it provides an opportunity for the OBA program to partner with other government ministries responsible for infrastructure.

The study found that managers and healthcare providers saw an impact of the voucher program on the accredited facilities and the targeted community. Majority of the providers and facility managers within the private sector felt that the voucher program is a profitable business model. The voucher program was also associated with increased income to the health facilities through the claims and reimbursement process, however this effect was reported more by the private sector compared to the public and faith based facilities. The revenues from the voucher program have been used by facility managers to upgrade the facilities, purchase drugs, supplies, equipment’s, provide incentives to clients such as improve hygiene and nutrition during admission, purchase of beds, provide incentives to providers to deliver quality services and compensate long working hours [24]. In the public facilities that were able to utilize the funds, the revenues have been utilized to improve health education on competition among health facilities in the region across the sectors which has led to an overall improvement of quality of care in the facilities. Private facilities appear to have used these revenues to expand and improve the quality of services as compared to public facilities. It was evident that the business model was more appreciated by the private and faith-based sector facilities compared to the public sector; this was mainly influenced by the facilities’ ability to make decisions on the utilization of the revenues. This perhaps implies the need of the County health departments within the devolved system to allow public facilities to utilize the funds to improve their facilities and make them competitive. This could be explained by the fact that privately owned facilities had the freedom to invest their funds in infrastructure, equipment, staff and client comfort, which satisfied and attracted more clients. In this setting, competition has been associated with improved quality of care and efficiency and suggest that results-based financing is associated with increased use and quality of care [9, 10, 20, 22].

The majority of the facility managers and providers felt that the voucher program increased access to services and reduced inequities by making RH services affordable to the poor. A population survey conducted in the same context to assess utilization of reproductive health services showed substantially higher proportions of voucher clients compared to poor non-voucher clients obtained LARC-PM, delivered at health facility and received skilled birth care and post-natal care services [25] This resonates with previous findings that demonstrate that voucher programs are associated with improving access and utilization of health services [21, 26]. By accrediting many health facilities at different levels, the voucher increased client choice and promoted competition among healthcare providers for the clients. The program’s competitiveness threatened the public facilities because given the freedom of choice by the voucher, many users had more confidence in, and utilized private facilities compared to public facilities.

Majority of facility managers and healthcare providers felt that the program was associated with a reduction of maternal and newborn mortality. There is need to conduct a prospective study that measures the impact of the voucher program on the reduction of maternal and neonatal deaths to verify this perception. In addition it was felt that the general health seeking behaviors of mother had improved. For example, majority of providers felt that mothers began attending ANC clinics early and that the proportion of mothers attending ANC clinics had significantly increased. It was also felt that the voucher had also led to increased facility deliveries within this setting. However, majority of providers felt that the vouchers had not been used to access PNC services compared to other services. Similar findings from other studies showing the association of voucher programs with increased facility deliveries and ANC uptake have been published [9, 19, 21, 23, 27]. A recent study published in the same context shows that PNC services were least known by women and that they reported very low PNC services [26]. The program needs to develop strategies to increase PNC uptake within this context.

## Conclusion

The study identified some key lessons. For the successful program functioning there is need to ensure that the benefit package and criteria for targeting beneficiaries are well understood by managers and healthcare providers in the accredited facilities. Moreover, continuous clinical and program related training is required to emphasize both the quality in-service delivery as well as administrative matters such as reimbursement and claims process.

Income earned through the voucher program can be used to improve quality of care to satisfy clients’ needs and ultimately improving health seeking behaviors of the target population. However within the public sector, there are constrains on utilizing such funds and there is a need to explore ways that allow the public sector facilities to directly use the funds to reflect their client’s needs. The sustainability of the voucher program is greatly influenced by the client volume for the participating providers and the facility readiness to provide the services. Services that are stigmatized and critical such as GBVRS are not well addressed by voucher programs. Services that are provided by a wide range of health facilities are better accessed and utilized compared to services that requires referral for specialized services.

In this context, the vouchers programs are increasingly acknowledged as a promising health financing mechanism enabling access and improvement of quality of health services.

### Limitations of the study

Although this study collected data from facilities of different size, ownership status and a both facility managers and healthcare provider, there were also limitations to acknowledge. First, as a qualitative study, the findings do not generalize beyond the interviewed providers and managers, although effort was made to draw from different experiences and settings. Secondly, the interviews may have been affected by the availability of the facility managers and healthcare providers; we conducted the interviews very early morning or in the evening when the client load at the facilities was low. Thirdly, societal attitudes and beliefs many not only affect service delivery but also determine the extent to which issues were discussed by respondents. Overall to strengthen dependability, different research assistants conducted the interviews and interview guides were used to make sure the interviews covered the same areas. Interviews were conducted until data saturation was reached.

### Adherence to RATS guidelines

We confirm that the paper adheres to RATS guidelines for reporting qualitative studies as outlined in *Qualitative Research Review Guidelines–RATS* available at: http://www.biomedcentral.com/ifora/rats. In particular, the research question, the study design, the criteria for selecting participants, and the analytical approach are explicitly described.

## Abbreviations

DSF: Demand-side health financing
OBA: Output-based aid
RH: Reproductive Health
GBVRS: Gender based violence recovery services
VMA: Voucher management agency
IDIs: In-depth interviews
CS: Caesarean section
ANC: Antenatal care
LARC: Long acting and reversible contraception
PM: Permanent methods
